# Obesity paradox and aging: Visceral Adiposity Index and all-cause mortality in older individuals: A prospective cohort study

**DOI:** 10.3389/fendo.2022.975209

**Published:** 2022-10-10

**Authors:** Lei Wang, Zhong Yi

**Affiliations:** ^1^ Department of Cardiology, Aerospace Center Hospital, Beijing, China; ^2^ Department of Geriatric Medicine, Aerospace Center Hospital, Beijing, China

**Keywords:** visceral adiposity index, all-cause mortality, older individuals, obesity paradox, aging

## Abstract

**Background:**

The relationship between body mass index (BMI) and mortality in older adults diminished. It is necessary to examine other factors that may accurately predict mortality in older adults. The visceral adiposity index (VAI) is an uncomplicated marker specific to the gender that incorporates anthropometric data and lipid profiles. VAI has been proposed as a marker of visceral adipose tissue dysfunction and of the related cardiometabolic risk. The aim of this study was to evaluate the link of VAI with all-cause mortality among the elderly.

**Methods:**

The present prospective cohort study included data from 1999 to 2014 provided by the National Health and Nutrition Examination Survey (NHANES) in the United States. NHANES participants at or above the age of 65 were included. Data collection was carried out by taking face-to-face interviews, mobile-physical examinations, and lab tests. From the start of the survey to the end of December 2015, mortality-related follow-up statistics are available. The shape of the link between VAI and all-cause mortality was investigated using a restricted cubic spline model. Univariate- and multivariate-adjusted Cox proportional hazard models were estimated for VAI, and the results were presented as regression coefficients and 95% confidence intervals (CI).

**Results:**

The 82,091 NHANES participants represented 442.2 million non-institutionalized residents of the United States. A total of 11,173 older individuals (representing 23.3 million; aged 73.4 ± 5.8 years; 56.3% women, 82.7% non-Hispanic Whites, 6.8% non-Hispanic Blacks, and 3.3% Mexican Americans) were included in the study. During the 80-month follow-up period, 4466 fatalities were reported, including 825 deaths from cancer, 867 from heart disease, and 211 from cerebrovascular disease. The restricted cubic spline model demonstrated a robust J-shaped link between VAI and all-cause mortality, revealing a significant decrease in risk within the lower range of VAI, which attained the lowest risk close to 1.7. With VAI greater than 1.7, the risk of mortality increased with the increase of VAI (P for non-linearity = 0.025). In the multivariate-adjusted model, the risk of all-cause mortality was 0.73 (0.56-0.97) and 1.05 (1.01-1.09) in participants with VAI less than 1.7 and VAI greater than or equal to 1.7, respectively.

**Conclusion:**

This investigation is a population-based cohort study with high sample sizes and a long-term in older individuals follow-up that showed a J-shaped link between VAI levels and all-cause mortality. Understanding the independent roles of VAI in the relationship between BMI and mortality is crucial to understanding the obesity paradox phenomenon.

## Background

In the United States as well as the rest of the world, obesity is a significant public health issue. The body mass index (BMI) is widely accepted as a good indication of general adiposity, and a number of epidemiological studies have demonstrated that obesity, as measured by BMI, is a substantial risk factor for a variety of long-term illnesses and death (1, 2). The link between BMI and mortality, on the other hand, has sparked a lot of debate, since epidemiological studies have discovered numerous forms of U-shaped, J-shaped, and linear relationships between BMI and mortality (3–8). In several studies, being overweight, for example, was linked to an increased risk of mortality (9), while others found that overweight persons had the lowest death rate and that mortality increased with decreased BMI (10, 11). The phenomenon has been described as “obesity paradox” (12). The relationship between BMI and mortality in older adults diminished. It is necessary to examine other factors that may accurately predict mortality in older adults. BMI is an imprecise measure of adiposity, which is a significant yet understudied methodological restriction in obesity research. Although BMI reflects overweight in relation to height, it does not take into account body composition. People with the same BMI have greatly varied compositions. This is critical since the position of fat depots may have diverse effects on health outcomes, including mortality.

The visceral adiposity index (VAI) is an uncomplicated marker specific to the gender that incorporates anthropometric data and lipid profiles to provide a reliable predictor of visceral dysfunction. The formula includes four parameters: waist circumference (WC), BMI, serum triglycerides (TG), and high-density lipoprotein cholesterol (HDL-C). The following equations are used to calculate VAI in men and women (13):

Male: VAI = (WC/(39.68 + 1.88*BMI)) * (TG/1.03) * (1.31/HDL-C)
Female: VAI = (WC/(36.58 + 1.89*BMI)) * (TG/0.81) * (1.52/HDL-C)
VAI, visceral adiposity index
WC, waist circumference (cm)
BMI, the body-mass index is determined as follows: the weight in kilograms (Kgs)/(height in square meters (m^2^)
TG, triglycerides (mmol/l)

HDL-C, high-density lipoprotein cholesterol (mmol/l)

VAI can serve as a useful index of both the distribution and function of fat (14). VAI was recently developed for the identification of visceral adiposity dysfunction. A significant link between VAI and cardiometabolic risk has been observed in several studies. Nusrianto et al. recently discovered that VAI is a practical formula for estimating visceral fat accumulation and may be used to predict the incidence of type 2 diabetes mellitus (T2DM) in the Asian population (15). Several studies have found that VAI is a predictor of incident hypertension (16, 17). According to Randrianarisoa et al., the calculation of VAI may provide a better estimation of subclinical atherosclerosis than calculating the homeostatic model evaluation of insulin resistance (HOMA-IR) (18).

According to several research findings, the link between body mass index (BMI) and older people’s mortality differs from that for younger adults. In older adults, a low BMI is linked to a higher chance of mortality. On the other hand, further studies are required to investigate the link between VAI and all-cause mortality among the older population. The objective of this research was to evaluate the link of VAI with the risk of all-cause mortality in older people.

## Methods

### Study design and population

The present prospective cohort study included the data provided by the National Health and Nutrition Examination Survey (NHANES) in the United States from 1999 to 2014. NHANES participants at or above the age of 65 were included. The NHANES adopted a complex, multistage, probability sampling method to collect the health data representing the US population. Data collection was done by taking face-to-face interviews, mobile-physical examinations, and lab tests.

### Baseline data collection

To collect data on covariates, baseline questionnaires were used, including gender, age, marital status, education level, race/ethnicity, family income-poverty ratio, smoking status, self-reported baseline medical history (diabetes, myocardial infarction, hypertension, hypercholesterolemia, cardiovascular disease, stroke, and chronic bronchitis), and medications (antihypertensives, hypoglycemic agents, and lipid-lowering medications). The height and weight measurements were utilized to determine the BMI. Laboratory measurements were performed in accordance with the laboratory procedure manual for NHANES. The NHANES operational instructions (https://wwwn.cdc.gov/nchs/nhanes/analyticguidelines.aspx) define the methodology and processes used for clinical laboratory data and study visits, correspondingly.

The diagnostic criteria for smoking are: never-smoked less than 100 cigarettes in their lives, former-smoked more than 100 cigarettes in their lives and no longer smoke, now-smoked more than 100 cigarettes in their lives and smoke some days or daily.

The diagnostic criteria for diabetes are (19): patient self-report of a diagnosis of diabetes, use of diabetes medication or insulin, hemoglobin A1c (HbA1c) level ≥ 6.5%, fasting plasma glucose ≥ 7.0 mmol/l (126 mg/dl), random plasma blood glucose ≥ 11.1 mmol/l (200 mg/dl), two-hour oral glucose tolerance test (OGTT) blood glucose ≥ 11.1 mmol/l(200 mg/dl).

The diagnostic criteria for hypertension are (20, 21): patient self-report of a diagnosis of hypertension, usage of antihypertensives medication, or average blood pressure (systolic blood pressure 140 mmHg and/or diastolic blood pressure ≥ 90 mmHg). Average blood pressure was calculated by the following protocol: The diastolic reading with zero is ignored when measuring the diastolic average. The zero value of all diastolic readings indicated zero as the average. If just one blood pressure reading was collected, that was the average reading. When there are many blood pressure readings, the first one is always excluded when calculating the average.

The cardiovascular disease (CVD) status of participants was established by a self-reported diagnosis of at least one of the five following CVD subtypes: coronary artery disease (CAD), congestive heart failure (CHF), angina, myocardial infarction, and stroke. Self-reported positive selection (yes or no) for at least one of these diseases was used to determine the existence of CVD, and CVD respondents might fall into more than one CVD subtype category.

According to the KDIGO 2021 Clinical Practice Guideline, chronic kidney disease (CKD) is described as an abnormality of kidney function (22).

Mortality

From 1999 to 2014, the NHANES-assigned sequence number was used to link de-identified and anonymized participant data to longitudinal Medicare and mortality data. Mortality follow-up statistics are provided from the start of survey participation until the end of December 2015. The all-cause mortality and mortality associated with heart diseases (I00–I09, I11, I13, I20–I51), Alzheimer’s disease (G30), diabetes mellitus (E10–E14), malignant neoplasms (C00–C97), chronic lower respiratory diseases (J40–J47), cerebrovascular diseases (I60–I69), and other causes were evaluated. The International Classification of Diseases (10^th^ edition) was utilized to determine the cause of death.

### Statistical analysis

Continuous variables are expressed as mean standard deviation (SD; Gaussian distribution) or median (range; Skewed distribution), while categorical variables are expressed as numbers (%). To test the differences among the VAI quartiles, χ2 (categorical variable), one-way ANOVA test (normal distribution), or Kruskal–Wallis H test (skewed distribution) were used. We utilized a restricted cubic spline model for examining the shape of the correlation between VAI and all-cause mortality. The three knots were chosen at the 25^th^, 50^th^, and 75^th^ quartiles. Before the data analysis, variables were evaluated for missing values. The proportion of missing data ranged from 0 to 45.9% (CKD medical history). Dummy variables were used to indicate missing covariate values for the purpose of including these data in the analysis. Missing covariate values of categorical variables were included as a group in multiple regression, and missing covariate values of continuous variables were included as a group in multiple regression after being treated as categorical variables (23).

Furthermore, univariate- and multivariate-adjusted Cox proportional hazard models for VAI were estimated, and the results were presented as regression coefficients and 95% confidence intervals (CI). Regression models were generated for the entire sample and adjusted for demographics, socioeconomics, behavior, anthropometric variables, and medical history.

### Sensitivity analysis

A restricted cubic spline model was used to assess the shape of the correlation between VAI and BMI and all-cause mortality in different sexes. The three knots were chosen at the 25^th^, 50^th^, and 75^th^ quartiles. Furthermore, univariate- and multivariate-adjusted Cox proportional hazard models for BMI were estimated, and the results were presented as regression coefficients and 95% confidence intervals (CI).

All statistical analyses were done employing the R package (The R Foundation; http://www.r-project.org; version 4.1.2) and were modified for complex survey design and population weighting following survey protocols. The findings may be applied and extrapolated to the entire adult population of the United States by incorporating population weights, stratum variables, and main sampling units into the analysis, accounting for differential probability of inclusion into the sample and non-response bias.

## Results

**Figure 1:**
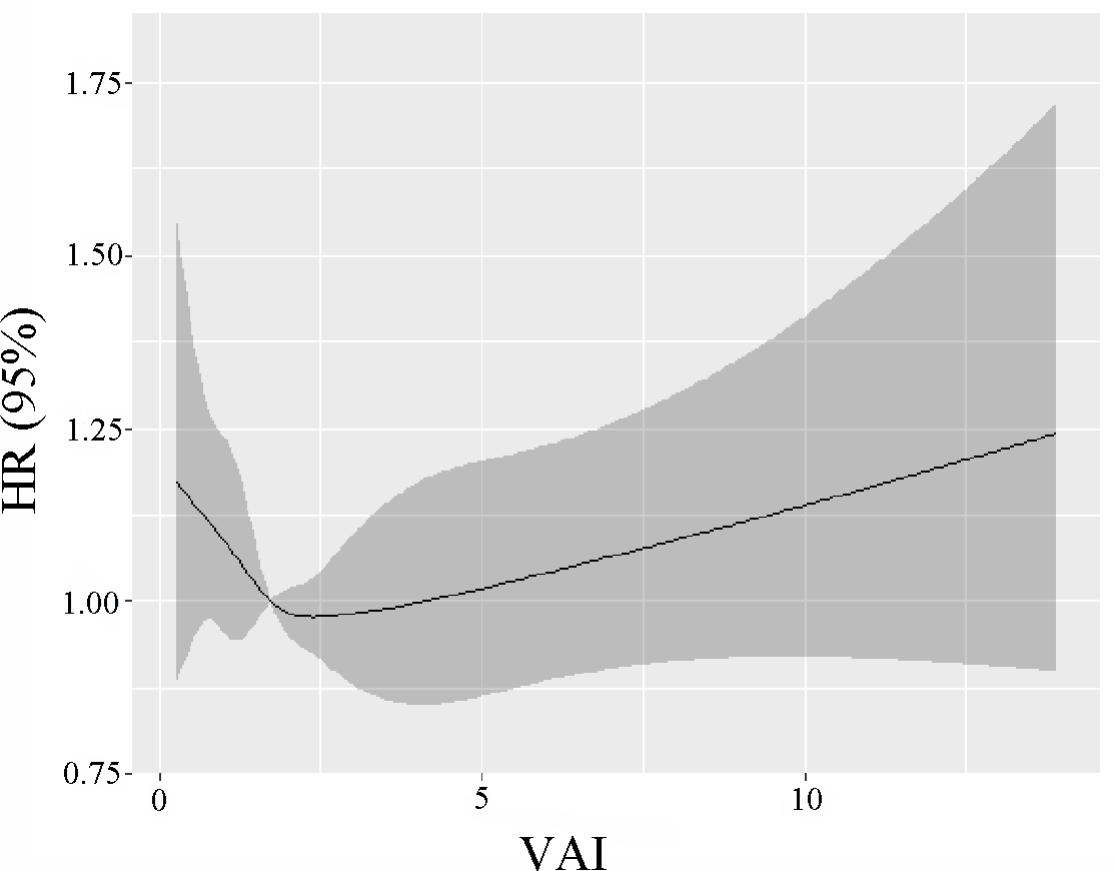
The unadjusted correlation between continuous VAI and all-cause mortality is demonstrated using generalized additive models.

The 82091 NHANES participants represented 442.2 million noninstitutionalized residents of the US. The study comprised a total of 11173 older individuals (representing 23.3 million; aged 73.4 ± 5.8 years; 56.3% women, 82.7% non-Hispanic Whites, 6.8% non-Hispanic Blacks, and 3.3% Mexican Americans). The absence of marital status differs between males and females (**Table S1**). **Table 1** demonstrates the weighted baseline features of study subjects, stratified by the quartiles of VAI. Between the quartiles of VAI, there was a significant age variation (P = 0.04). The people in the second quartiles are older (74.0 ± 6.0 years). In the fourth quartiles, the female ratio was high, whereas the male ratio was high in the first quartiles (P < 0.001). Most of the Non-Hispanic Whites were in the fourth quartile (84.7%, P < 0.001). In the first quartiles, the married rate was high (63.1%, P = 0.44). In this study, most participants with a “college or above” level were found in the first quartiles (54.8%, P < 0.001). There were 4466 deaths reported during the 80-month study, including 825 deaths from cancer, 867 from heart disease, and 211 deaths from cerebrovascular disease. **Figure 1** shows how restricted cubic splines were used to create a flexible model and describe the unadjusted correlation between VAI and all-cause mortality. J-shaped link of VAI with all-cause mortality revealed a significant decrease in risk in the lower range of VAI, which attained the lowest risk close to 1.7. With VAI greater than 1.7, the probability of death increased with the increase of VAI (P for non-linearity = 0.025). **Table 2** presents the unadjusted and adjusted models for the risk of all-cause mortality. Within the weighted unadjusted model, the risk of all-cause mortality was 0.86 (0.65-1.14) and 1.04 (0.99-1.10) in participants with VAI <1.7 and VAI ≥ 1.7, respectively. In model 5, the risk of all-cause mortality was 0.73 (0.56-0.97) and 1.05 (1.01-1.09) in participants with VAI <1.7 and VAI ≥ 1.7, respectively, after adjusting for demographics, socioeconomics, behavior, anthropometric variables, medical history, medications, and laboratory findings.

**Table 1:** Weighted characteristics of the study participants by the quartiles of VAI.

	Level	Overall	Q1	Q2	Q3	Q4	*p*
VAI			< 1.10	1.10 - 1.69	1.70 - 2.79	≥2.80	
N		23285519	5606699	5313948	6221989	6142883	
Age (mean (SD))		73.4 (5.8)	73.5 (5.9)	74.0 (6.0)	73.3 (5.6)	72.9 (5.8)	0.04
Sex (%)	Female	13110334 (56.3)	2514671 (44.9)	2949939 (55.5)	3776827 (60.7)	3868898 (63.0)	<0.001
	Male	10175185 (43.7)	3092029 (55.1)	2364009 (44.5)	2445163 (39.3)	2273985 (37.0)	
Race/ethnicity (%)	Mexican American	778769 (3.3)	123930 (2.2)	176451 (3.3)	220298 (3.5)	258090 (4.2)	<0.001
	Non-Hispanic Black	1583887 (6.8)	622153 (11.1)	390917 (7.4)	379183 (6.1)	191635 (3.1)	
	Non-Hispanic White	19254071 (82.7)	4540857 (81.0)	4432917 (83.4)	5079494 (81.6)	5200804 (84.7)	
	Other Race	1668792 (7.2)	319759 (5.7)	313663 (5.9)	543015 (8.7)	492355 (8.0)	
Education (%)	College or above	10683228 (45.9)	3071028.6 (54.8)	2560692 (48.2)	2760479 (44.4)	2291028 (37.3)	<0.001
	High school or equivalent	6426240 (27.6)	1221594 (21.8)	1489826 (28.0)	1694387 (27.2)	2020434 (32.9)	
	Less than high school	6143023 (26.4)	1310054 (23.4)	1255337 (23.6)	1753017 (28.2)	1824614 (29.7)	
Marital status (%)	Married	13964109 (60.0)	3539173 (63.1)	3135788 (59.0)	3672492 (59.0)	3616656 (58.9)	0.44
	Never married	885308 (3.8)	238119 (4.2)	205958 (3.9)	179646 (2.9)	261586 (4.3)	
	Separated	8017205 (34.4)	1769205 (31.6)	1880278 (35.4)	2217576 (35.6)	2150146 (35.0)	
Family income-poverty ratio (mean (SD))		2.76 (1.49)	3.06 (1.55)	2.78 (1.49)	2.64 (1.46)	2.58 (1.43)	<0.001
Family income-poverty ratio (%)	<1.0	2150186 (9.2)	425693 (7.6)	394844 (7.4)	628191 (10.1)	701459 (11.4)	<0.001
	1.0-3.0	10423339 (44.8)	2115258 (37.7)	2473771 (46.6)	3022065 (48.6)	2812245 (45.8)	
	>3.0	8424720 (36.2)	2434080 (43.4)	2011887 (37.9)	1964729 (31.6)	2014023 (32.8)	
Smoking status (%)	Never	11088629 (47.6)	2594945 (46.3)	2519057 (47.4)	3040680 (48.9)	2933946 (47.8)	0.138
	Former	10044131 (43.1)	2618102 (46.7)	2345428 (44.1)	2524576 (40.6)	2556026 (41.6)	
	Now	2138547 (9.2)	392147 (7.0)	449463 (8.5)	654408 (10.5)	642530 (10.5)	
Diabetes (%)	No	16634205 (71.4)	4652827 (83.0)	3877194 (73.0)	4421329 (71.1)	3682856 (60.0)	<0.001
	Yes	6651314 (28.6)	953872 (17.0)	1436754 (27.0)	1800660 (28.9)	2460028 (40.0)	
Hypertension (%)	No	9651068 (41.4)	2638862 (47.1)	2304467 (43.4)	2461528 (39.6)	2246211 (36.6)	0.001
	Yes	13634452 (58.6)	2967837 (52.9)	3009481 (56.6)	3760461 (60.4)	3896672 (63.4)	
CVD (%)	No	16970868 (72.9)	4384955 (78.2)	3707784 (69.8)	4488395 (72.1)	4389734 (71.5)	0.057
	Yes	6313676 (27.1)	1221744 (21.8)	1605189 (30.2)	1733595 (27.9)	1753149 (28.5)	
CKD (%)	No	7511966 (32.3)	2438885 (43.5)	1770781 (33.3)	1852014 (29.8)	1450286 (23.6)	<0.001
	Yes	4500691 (19.3)	1097486 (19.6)	1018028 (19.2)	1229365 (19.8)	1155812 (18.8)	

VAI, visceral adiposity index; CVD, cardiovascular disease; CKD, chronic kidney disease. Data are presented as frequencies (percentages) or mean (SD) or median (IQR). Numbers that do not add up to 100% are attributable to missing data.

**Table 2:** Weighted associations between the VAI and all-cause mortality in the multivariable, and crude analyses.

VAI	<1.7	≥ 1.7
	HR (95%CI) p	HR (95%CI) p
Model 1	0.86 (0.65-1.14) 0.300	1.04 (0.99-1.10) 0.100
Model 2	0.92 (0.70-1.20) 0.530	1.05 (1.00-1.10) 0.064
Model 3	0.84 (0.63-1.10) 0.200	1.05 (1.01-1.10) 0.023
Model 4	0.81 (0.61-1.09) 0.171	1.05 (1.01-1.10) 0.028
Model 5	0.73 (0.56-0.97) 0.029	1.05 (1.01-1.09) 0.031

HR, hazard ratio, CI, confidence interval; VAI, visceral adiposity index; Model 1, unadjusted; Model 2, adjusted for age; Model 3, adjusted for age, sex; Model 4, adjusted for model 3 covariates plus ethnicity, family income-poverty ratio level, education, and marital status; Model 5, adjusted for model 4 covariates plus smoking status, diabetes, hypertension, CVD, and CKD. Data are hazard ratio (95% CI).

### Sensitivity analysis

A strong J-shaped relationship between VAI and all-cause mortality in the different sex was observed (**Figure S1**). The correlation shape between BMI and all-cause mortality in different sexes revealed that overweight people had the lowest mortality and that this mortality tended to increase as BMI decreased (**Figure S2**). The unadjusted and adjusted models for BMI and the all-cause mortality risk are presented in **Tables S2**, **S3**. After adjusting for demographics, socioeconomics, behavior, anthropometric variables, medical history, medications, and laboratory findings, the risk of all-cause mortality was 0.97 (0.97-1.00) in model 5.

## Discussion

In this population-based cohort study, a J-shaped link between VAI levels and all-cause mortality was discovered, which included large sample sizes as well as a long-term follow-up in older individuals. The VAI is a more specific and sensitive examination tool than the BMI. These results provide a concrete solution to the obesity paradox.

Recently, Lee et al. found that increased visceral-to-subcutaneous fat area ratio (VSR), was a reliable predictor of all-cause mortality (24). This shows that the position of fat deposits matters more than the actual amount of fat present in the body. Many studies suggest that as people grow older, central fat and fat-free mass reduction may become more important than BMI in shaping the health risks associated with obesity (25). Some studies show that the VAI is a reliable indicator of increased patient risk for cardiometabolic diseases (26–29), especially in elderly women (30). Recent data have highlighted abdominal obesity, as measured by waist circumference, as a risk marker for cardiovascular disease that is independent of BMI. Imaging methods for assessing body composition, particularly visceral adiposity, have also advanced significantly. According to studies that measure fat depots, including ectopic fat, excess visceral adiposity is a self-regulating indication of poor cardiovascular outcomes (31).

The study found that older overweight persons had the lowest death rate and that mortality increased with decreased BMI. It may not be appropriate to use only BMI to predict obesity and associated cardiometabolic diseases. Inaccurate evaluations may result in a systematic underestimation of obesity’s influence on morbidity and premature mortality. The excess fat mass has shown to be harmful to one’s health while accumulating research suggests that fat distribution has an effect on one’s health. Therefore, understanding the distribution of fat might result in the detailed understanding of the obesity paradox as well as important clinical and public health messages about healthy body composition beyond BMI. Deepened knowledge regarding changes in body composition and fat distribution will help researchers better anticipate the link between obesity, illness, and mortality among older people (25, 32). A J-shaped link between VAI levels and all-cause mortality was discovered. VAI can serve as a significant index for fat distribution and function (14).

### Limitations

The current study had various limitations because this was an observational study and did not have available data on behavioral (nutrition, physical activity, sleep, and frailty measures) changes. Also, only all-cause mortality was evaluated. The relationship between other prognostic indicators and VAI requires further study.

## Conclusion

This is a population-based cohort study incorporating large sample sizes and a long-term follow-up in older individuals identifying a J-shaped link between VAI and all-cause mortality levels. Understanding the independent roles of VAI in the relationship between BMI and mortality can help to explain the obesity paradox phenomena.

## Data availability statement

The datasets presented in this study can be found in online repositories. The names of the repository/repositories and accession number(s) can be found in the article/**Supplementary Material**.

## Ethics statement

The studies involving human participants were reviewed and approved by The National Center for Health Statistics (NCHS) Research Ethics Review Board. The patients/participants provided their written informed consent to participate in this study.

## Author contributions

ZY and LW made contributions to data collection and drafted the manuscript. ZY and LW made contributions to the analysis and interpretation of the data. All authors reviewed the manuscript. All authors contributed to the article and approved the submitted version.

## Conflict of interest

The authors declare that the research was conducted in the absence of any commercial or financial relationships that could be construed as a potential conflict of interest.

## Publisher’s note

All claims expressed in this article are solely those of the authors and do not necessarily represent those of their affiliated organizations, or those of the publisher, the editors and the reviewers. Any product that may be evaluated in this article, or claim that may be made by its manufacturer, is not guaranteed or endorsed by the publisher.
