# Supplementary material for: Obesity paradox and aging: Visceral Adiposity Index and all-cause mortality in older individuals: A prospective cohort study
Source: Front Endocrinol (Lausanne). 2022 Oct 10;13:975209. doi: 10.3389/fendo.2022.975209 (PMC9589042; doi:10.3389/fendo.2022.975209)
Supplement: Supplementary file 1 [file DataSheet_1.doc]

Supplemental Material

## Table S1. Weighted baseline characteristics of participants: VAI and VAI missing.

|  | Level | Overall | VAI | VAI missing | *p* |
| --- | --- | --- | --- | --- | --- |
| N |  | 54935673 | 23285519 | 31650154 |  |
| Age (mean (SD)) |  | 73.8 (6.0) | 73.4 (5.8) | 74.1 (6.0) | <0.001 |
| Sex (%) | Female | 31309431 (57.0) | 13110334 (56.3) | 18199097 (57.5) | 0.279 |
|  | Male | 23626242 (43.0) | 10175185 (43.7) | 13451057 (42.5) |  |
| Race/ethnicity (%) | Mexican American | 1733122 (3.2) | 778769 (3.3) | 954353 (3.0) | 0.006 |
|  | Non-Hispanic Black | 4403846 (8.0) | 1583887 (6.8) | 2819959 (8.9) |  |
|  | Non-Hispanic White | 44810948 (81.6) | 19254071 (82.7) | 25556877 (80.7) |  |
|  | Other Race | 3987758 (7.3) | 1668792 (7.2) | 2318966 (7.3) |  |
| Education (%) | College or above | 24123564 (43.9) | 10683228 (45.9) | 13440337 (42.5) | 0.006 |
|  | High school or equivalent | 14933343 (27.2) | 6426240 (27.6) | 8507103 (26.9) |  |
|  | Less than high school | 15731573 (28.6) | 6143023 (26.4) | 9588550 (30.3) |  |
| Marital status (%) | Married | 31018974 (56.5) | 13964109 (60.0) | 17054865 (53.9) | <0.001 |
|  | Never married | 2213128 (4.0) | 885308 (3.8) | 1327820 (4.2) |  |
|  | Separated | 20506330 (37.3) | 8017205 (34.4) | 12489124 (39.5) |  |
| Family income-poverty ratio (mean (SD)) |  | 2.68 (1.49) | 2.76 (1.49) | 2.62 (1.49) | 0.011 |
| Family income-poverty ratio (%) | <1.0 | 5840574 (10.6) | 2150186 (9.2) | 3690388 (11.7) | 0.065 |
|  | 1.0-3.0 | 24840399 (45.2) | 10423339 (44.8) | 14417060 (45.6) |  |
|  | >3.0 | 18935568 (34.5) | 8424720 (36.2) | 10510848 (33.2) |  |
| Smoking status (%) | Never | 27107760 (49.3) | 11088629 (47.6) | 16019131 (50.6) | 0.054 |
|  | Former | 22787962 (41.5) | 10044131 (43.1) | 12743831 (40.3) |  |
|  | Now | 4958580 (9.0) | 2138547 (9.2) | 2820033 (8.9) |  |
| Diabetes (%) | No | 41091097 (74.8) | 16634205 (71.4) | 24456891 (77.3) | <0.001 |
|  | Yes | 13844577 (25.2) | 6651314 (28.6) | 7193263 (22.7) |  |
| Hypertension (%) | No | 21887856 (39.8) | 9651068 (41.4) | 12236789 (38.7) | <0.001 |
|  | Yes | 33047817 (60.2) | 13634452 (58.6) | 19413365 (61.3) |  |
| CVD (%) | No | 39431375 (71.8) | 16970868 (72.9) | 22460507 (71.0) | 0.111 |
|  | Yes | 15498214 (28.2) | 6313676 (27.1) | 9184538 (29.0) |  |
| CKD (%) | No | 15169384 (27.6) | 7511966(32.3) | 7657418 (24.2) | <0.001 |
|  | Yes | 10869811 (19.8) | 4500691 (19.3) | 6369120 (20.1) |  |

Abbreviations:

VAI, visceral adiposity index

CVD, cardiovascular disease

CKD, chronic kidney disease

Table S2. Weighted univariate cox regression model

|  | Level | HR | CI | P |
| --- | --- | --- | --- | --- |
| Age |  | 1.10 | 1.09-1.12 | 0.000 |
| Sex | Female | 1 |  |  |
|  | Male | 1.36 | 1.22-1.51 | 0.000 |
| Race/ethnicity | Mexican American | 1 |  |  |
|  | Non-Hispanic Black | 1.32 | 1.03-1.70 | 0.030 |
|  | Non-Hispanic White | 1.29 | 1.06-1.57 | 0.012 |
|  | Other Race | 1.13 | 0.79-1.61 | 0.496 |
| Education | College or above | 1 |  |  |
|  | High school or equivalent | 1.24 | 1.05-1.46 | 0.011 |
|  | Less than high school | 1.36 | 1.15-1.61 | 0.000 |
| Marital status | Married | 1 |  |  |
|  | Never married | 0.97 | 0.69-1.36 | 0.870 |
|  | Separated | 1.40 | 0.77-1.57 | 0.000 |
| Family income-poverty ratio |  | 0.88 | 0.84-0.92 | 0.000 |
| Family income-poverty ratio (%) |  |  |  |  |
|  | <1.0 | 1 |  |  |
|  | 1.0-3.0 | 0.80 | 0.65-0.99 | 0.041 |
|  | >3.0 | 0.61 | 0.49-0.75 | 0.000 |
| VAI |  | 1.02 | 0.98-1.06 | 0.260 |
| BMI |  | 0.97 | 0.95-0.98 | 0.000 |
| Smoking status | Never | 1 |  |  |
|  | Former | 1.23 | 1.07-1.41 | 0.004 |
|  | Now | 1.53 | 1.23-1.91 | 0.000 |
| Diabetes |  | 1.40 | 1.20-1.62 | 0.000 |
| Hypertension |  | 1.02 | 0.88-1.20 | 0.760 |
| CVD |  | 1.95 | 1.65-2.31 | 0.000 |
| CKD |  | 2.69 | 2.19-3.31 | 0.000 |

Abbreviations:

VAI, visceral adiposity index

BMI, the body-mass index is determined as follows: the weight in kilograms (Kgs) / (height in square meters (m^2^)

CVD, cardiovascular disease

CKD, chronic kidney disease

Table S3. Weighted associations between the BMI and all-cause mortality in the multivariable, and crude analyses.

| BMI | HR (95%CI) p |
| --- | --- |
| Model1 | 0.97 (0.95-0.98) 0.000 |
| Model2 | 0.97 (0.95-0.98) 0.000 |
| Model3 | 0.99 (0.98-1.00) 0.120 |
| Model4 | 0.99 (0.98-1.00) 0.087 |
| Model5 | 0.97 (0.97-1.00) 0.018 |

Data are hazard ratio (95% CI)

Abbreviations:

HR, hazard ratio

CI, confidence interval

BMI, the body-mass index is determined as follows: the weight in kilograms (Kgs) / (height in square meters (m^2^)

Model 1 unadjusted.

Model 2 adjusted for age

Model 3 adjusted for age, sex.

Model 4 adjusted for model 3 covariates plus ethnicity, family income-poverty ratio level, education, and marital status.

Model 5 adjusted for model 4 covariates plus smoking status, diabetes, hypertension, CVD, and CKD.


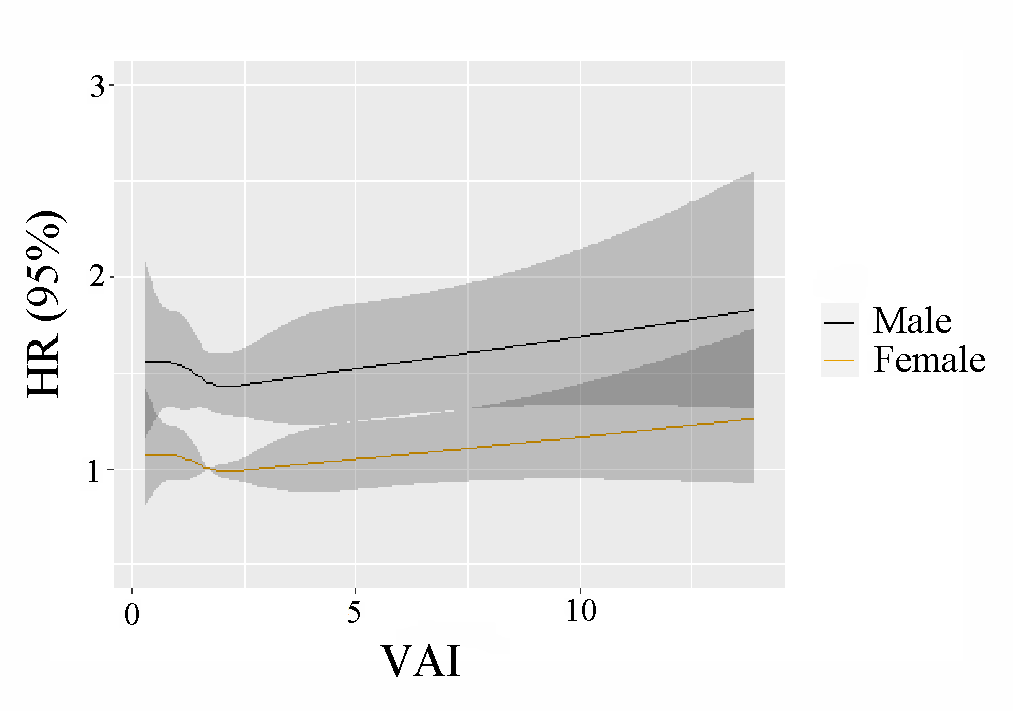


Figure S1: The correlation between continuous VAI and all-cause mortality is demonstrated using generalized additive models.


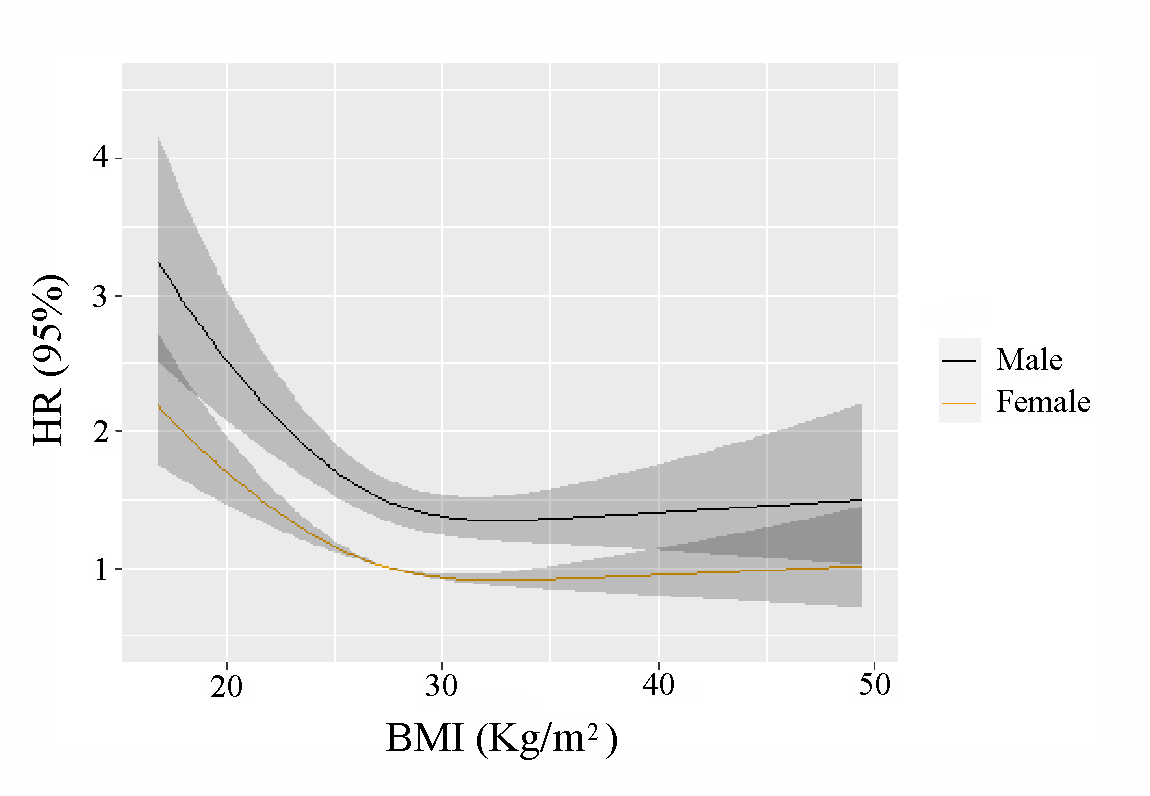


Figure S2: The correlation between continuous BMI and all-cause mortality is demonstrated using generalized additive models.
